# Per- and Polyfluoroalkyl Substances (PFAS): History, Current Concerns, and Future Outlook

**DOI:** 10.3390/molecules30224415

**Published:** 2025-11-14

**Authors:** Ryuichi Mashima

**Affiliations:** Department of Clinical Laboratory Medicine, National Center for Child Health and Development, 2-10-1 Okura, Setagaya-ku, Tokyo 157-8535, Japan; mashima-r@ncchd.go.jp; Fax: +81-3-3417-2238

**Keywords:** legacy PFAS, emerging PFAS, extractable organic fluoride, total oxide precursor

## Abstract

Per- and polyfluoroalkyl substances (PFAS) involve multiple per- and polyfluorinated compounds that are widely used globally. Legacy PFAS, including PFOA, PFOS, and PFHxS, were manufactured before 2000 in various industrialized nations, then gradually phased out in accordance with the Stockholm Convention. Due to the substantial accumulation of these legacy PFAS compounds, their concentrations in drinking water are regulated in some countries. This review first summarizes the historical background of PFAS, followed by a description of their chemical properties. The clinical manifestations of legacy PFAS in humans, such as dyslipidemia, attenuated immune function, and chronic kidney disorders, are also summarized. Emerging PFAS involve Gen-X and F-53B as well as numerous newly developed chemicals with their associated precursors/metabolites, including volatile PFAS. Research on these emerging PFAS compounds in the environment continues to grow, building a substantial body of evidence about their effects. The chemical structure of emerging PFAS shows a wide variety: they could contain ether, ester, sulfoneamide, and other halogen atoms rather than fluorine. Volatile PFAS involve the fluorotelomer 6:2 FTOH and other short-chain PFAS compounds, which are best measured by GC-MS. This review also briefly summarizes the assay for total oxidizable precursors of PFAS, an LC-MS-based assay for an emerging assay that will be used for a quantitative estimation of total PFAS, including emerging PFAS.

## 1. Introduction

### 1.1. History

**Figure 1:**
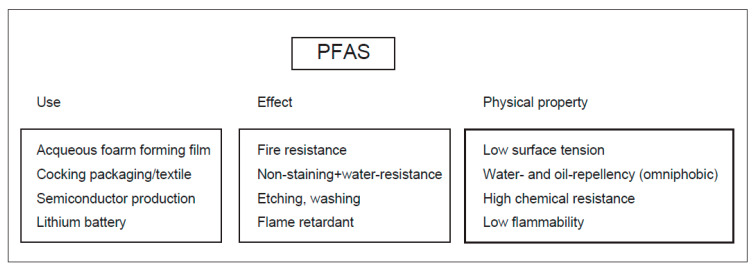
The uses, effects, and physical properties of PFAS.

**Figure 2:**
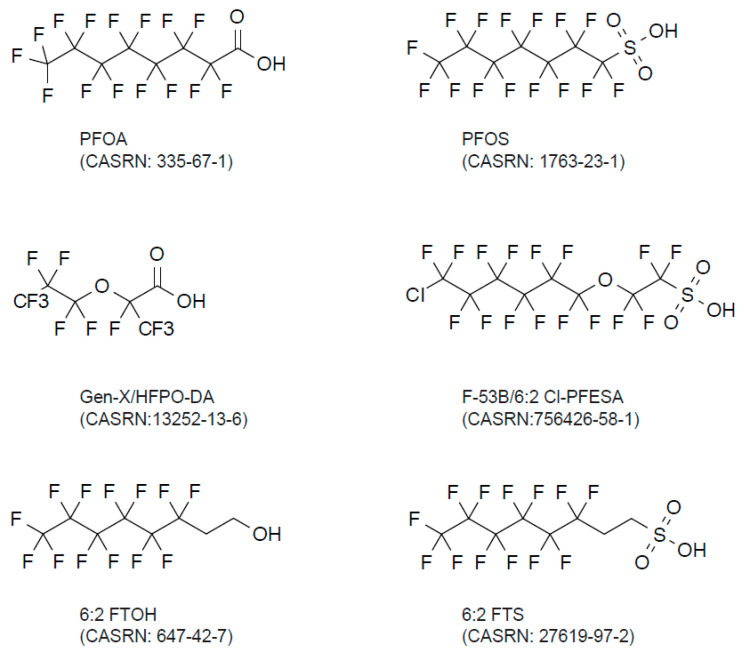
Chemical structure of PFAS.

Per- and polyfluoroalkyl substances (PFAS) have been used in a wide variety of applications, such as aqueous film-forming foam (AFFF), nonstick cookware, rubber packing materials, water-repellent fabrics, food packaging, cosmetics, and pesticides [1,2,3] (Figure 1). In 1938, Teflon (R), a prototypical PFAS, was invented by DuPont. PFAS are less susceptible to chemical degradation; thus, they are called “forever chemicals.” PFAS are an anthropogenic group of chemicals that are routinely detected in many industrialized countries in almost all samples (Table 1). Before 2000, a variety of PFAS compounds were produced. After the early 2000s, some major PFAS were phased out. Specifically, perfluorooctane sulfonate (PFOS, Figure 2) was first listed in the Stockholm Convention in 2009. Subsequently, perfluorooctanic acid (PFOA) was added in 2019, followed by perfluorohexane sulfonate (PFHxS) in 2022. In 2025, many industrialized countries followed this proposal [3].

This review article aims to overview the industrial history and chemical application of PFAS, as well as its remediation technique to conserve the global environment. Currently available data for remediation and health concerns are almost limited to legacy PFAS. The author wishes to summarize such legacy-PFAS-related data first, then expand the discussion for emerging PFAS-related issues. Furthermore, for the research paper on PFAS distribution, the geological fairness of the US, the EU, Asia, and the rest of the world was considered. The current concentration of emerging PFAS is still relatively low in soil, water, and atmosphere. However, an increasing number of studies raises a concern for the conversion to volatile PFAS from legacy PFAS. Thus, the author’s motivation will be focused on the environmental and biological outcome of currently producing emerging PFAS around the globe. This is a narrative review of published articles found in the PubMed database (Section 1, Section 2, Section 3 and Section 4). The cited articles of Section 5 (Perspectives) were selected from articles published in PubMed during the last 5 years.

### 1.2. Nomenclature: Legacy PFAS and Emerging PFAS

#### 1.2.1. Legacy PFAS

**Figure 3:**
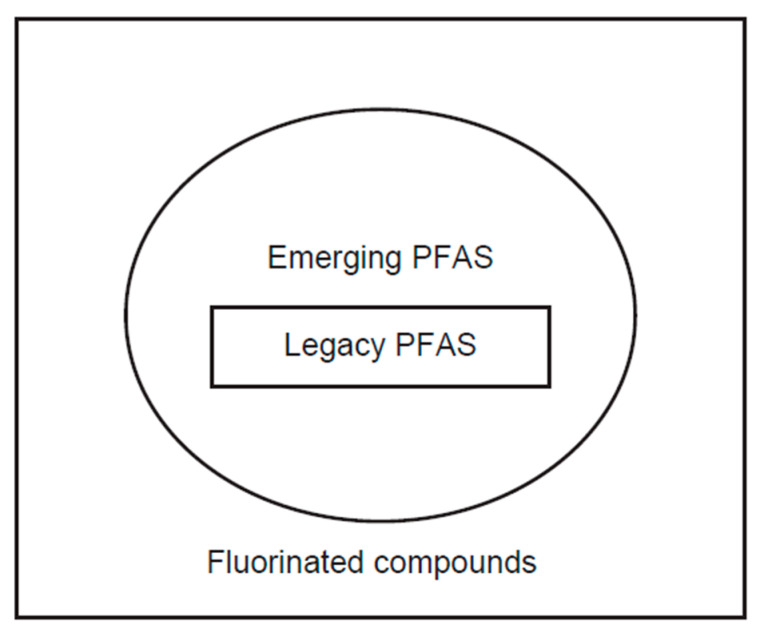
Legacy PFAS, emerging PFAS, and fluorinated compounds.

Currently, more than thousands of PFAS chemicals have been synthesized. While the term ‘legacy PFAS’ was not officially defined, this term itself is commonly found in recent publications [4,5,6,7,8,9,10,11] (Figure 3). It contains PFOA, PFOS, and several similar compounds with different chain lengths, such as PFUnDA (C10), PFNA (C9), PFHpA (C7), PFHxA (C6), and PFPA (C5). These well-characterized series of legacy PFAS species are routinely quantified using liquid chromatography–tandem mass spectrometry (LC-MS/MS) [12] (Table 1).

**Table 1:** Quantification procedure for PFASs.

Method	Target	Sample	Reference
LC-MS	Legacy PFAS	Food	[13]
	Legacy PFAS	Food	[14]
	Legacy PFAS	Food	[15]
	Legacy PFAS	Drinking water, river water, sea water	[16]
	Legacy PFAS	Human serum	[17]
GC-MS	Volatile PFAS	Indoor air	[18]
LC-Q-TOF	Non-target	Food	[15]
GC-Q-TOF	Volatile PFAS	Water	[19]
EOF	Total fluoride	Soil	[20]
TOP + EOF	Total fluoride	Honeybees and bee-collected pollen	[21]
	Total fluoride	Sugarcane pulp, tableware	[22]
	Total fluoride	Outdoor textiles, paper packaging, carpeting, and permanent baking sheets	[23]
	Total fluoride	Ski wax, snowmelts, and soil from skiing areas	[24]
	Total fluoride	Pooled human serum	[25]
	Total fluoride	Dust	[26]
	Total fluoride	Cosmetics	[27]

EOF, extractable organic fluoride; GC, gas chromatography; LC, liquid chromatography; MS, mass spectrometry; Q, quadrupole; TOP, total oxidizable precursors.

#### 1.2.2. Emerging PFAS

Emerging PFAS is another widely used term for newly developed PFAS compounds and their precursors/metabolites that are not included in legacy PFAS [1]. Recent evidence has demonstrated that these emerging PFAS are now widely detectable (reviewed in [28,29]) (Table 2). Emerging PFAS include Gen-X (hexafluoropropylene oxide-dimer acid, also known as HFPO-DA) and F-53B (2-((6-chloro-1,1,2,2,3,3,4,4,5,5,6,6-dodecafluorohexyl)oxy)-1,1,2,2-tetrafluoroethanesulfonic acid) [30] (Figure 2). Gen-X constitutes a common short-chain PFAS alternative, replacing PFOA, the linear long-chain PFOA representing the most common PFAS. F-53B is an alternative to the PFOS used in the industry, offering wide applications in manufacturing. These emerging PFAS usually contain, but are not limited to, an ether group [31]. 6:2 FTOH and 6:2 FTS are fluorotelomer PFAS, also classified as emerging PFAS. Based on these structures, there are also many telomer-structured emerging PFAS that might contain amide, ester, branched alkane, alkene, halogen atom, or other functional groups. In these cases, each perfluorinated compound, such as 6:2 FTOH, has a shorter carbon chain (C ≤ 6 in PFOS) [32]. Some such short-chained emerging PFAS are also called volatile PFAS; they are better quantified using gas chromatography–mass spectrometry (GC-MS) than LC-MS (Table 1). These shorter-chain PFAS accumulate in the air as the decomposition product of parental emerging PFAS by either chemical or biological pathways [2]. The biological effects of the emerging PFAS appeared to be similar [33]. The pharmacokinetics of emerging PFAS’ excretion from the human body have yet to be established.

##### Gen-X and F-53B

These are well-characterized emerging PFAS compounds with a chemical nature [33] (Figure 2). Although Gen-X and F-53B are recently emerging PFAS, they are already detectable globally. The quantification of Gen-X and F-53B was performed in LC-MS/MS (Table 2). These data demonstrate that the concentrations of Gen-X and F-53B are relatively lower than those of legacy PFAS, such as PFOS. It seems too early to discuss the biological effects of Gen-X and F-53 due to their small accumulation in humans. Furthermore, the inability to assess the authentic effects of Gen-X and F-53B in humans only allows us to estimate them for ethical reasons. This is due to widespread legacy PFAS contamination that cannot be properly controlled for in epidemiological studies.

##### Volatile PFAS

Volatile PFAS, such as PFBA, have attracted much attention [18]. How these volatile PFAS might have a deteriorating effect on humans remains unestablished. This is partly due to the quantification procedures used for these volatile PFAS. These concentrations should be carefully determined; otherwise, some or most PFAS might be lost during sample collection, storage, and pretreatment before analysis.

We might have an additional understanding of emerging PFAS substances when PFAS mitigation has been employed. Electrostatic interactions between carbons and fluoride in emerging PFAS are similarly found in legacy PFAS; thus, the bioremediation of emerging PFAS seems to undergo nearly similar efficiency. However, because they have a distinct structure from legacy PFAS, emerging PFAS are less likely to be microbiologically processed than PFOS.

### 1.3. PFAS Synthesis

The synthesis of PFAS has two separate methods [1]. One involves electrochemical fluorination, which replaces all hydrogen with fluoride in an electrochemical manner. This method has a high efficiency of fluoride replacement, while a branched PFAS has also been obtained at a high yield [40]. Another method involves the fluorotelomeric method, which allows higher efficiency of linear PFAS [41]. Terminal iodine acts as a hub for further reactions, such as hydroxylation and carboxylation.

### 1.4. PFAS Quantification

#### 1.4.1. LC-MS

**Figure 4:**
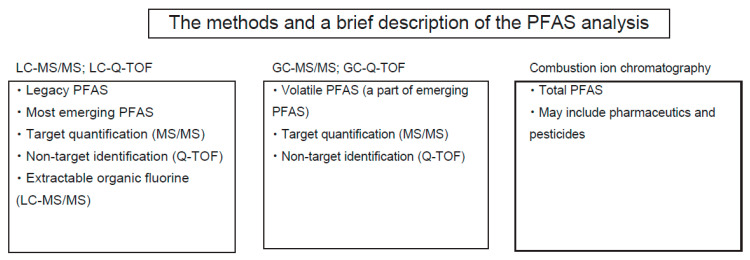
Analytical methods for PFAS.

LC-MS is a widely used quantitative platform for PFAS species for legacy PFAS with C ≥ 4 compounds [12] (Table 1) (Figure 4). For quantification, reversed-phase chromatography with MS/MS detection is employed. This assay has been used for a variety of specimens, such as soil, drinking water, food, serum, and plasma of humans and animals. Among the many legacy PFAS compounds, PFOS, PFOA, and PFHxS are routinely quantified for biomonitoring. In the U.S., the EU, and some other industrialized countries, such as Canada and Australia, their regulatory authorities now request the monitoring of legacy PFAS in drinking water [42,43]. Some other countries, such as Japan, will follow this trend in the coming year [42]. For PFAS quantification in water, two international standards (ISO 21675:2019, EPA 533/537.1/1633) are effective [16,44].

For the pretreatment of samples, particularly food, the QuEChERS (Quick, Easy, Cheap, Effective, Rugged, Safe) method is a sophisticated version of the multistep solid-phase extraction method developed by the United States Food and Drug Administration [13,14,15]. Currently, these services are commercially provided by multiple vendors. Essentially, a liquified sample was mixed with methyl tert-butyl ether, followed by the addition of MgSO_4_ as the desiccant. Collection of an organic layer allows a specimen to be ready for 1st solid-phase extraction, such as graphite carbon chromatography. Flow-through was collected and applied to a second solid-phase extraction, typically composed of weak anion exchange chromatography. Because most PFAS, such as PFOS and PFOA, are negatively charged at neutral pH, the elution of PFAS is achieved by an acidified solution to protonate a weak anion exchange resin. The collected eluate was then dried and concentrated when necessary, followed by an injection onto LC-MS/MS.

Each PFAS peak was separated using a C18 column and quantified by LC-MS/MS. It is well known that some species of bile acid comigrate with PFAS, leading to incorrect measurement of some PFAS [45,46]. For analytical laboratory consumables, a septum made with PFTE should be avoided because it generates contamination of fluorinated compounds. In cases where an unstable blank baseline of LC-MS/MS was observed, the insertion of a short trap column with the same packing materials as the analytical column between the upstream of the analytical column effectively eliminates contaminating PFAS for a high background.

The total oxidizable precursor (TOP) assay has been developed to estimate the levels of total PFAS, which is an ammonium persulfate-oxidizable PFAS, using LC-MS/MS [47,48]. Under this reaction, perfluorosulfuric acid (PFSA) undergoes perfluorocarboxylic acid (PFCA) via a hydroxyl radical-mediated reaction. An increasing number of studies have reported PFAS data using the TOP assay, suggesting that an increase in the role of emerging PFAS in the environment and humans needs to be properly assessed in the future.

#### 1.4.2. GC-MS

GC-MS is normally used for quantification of volatile PFAS, short-chained PFAS (C ≤ 4) species with high volatility [6,49] (Table 1). For enrichment of PFAS analytes, headspace solid-phase microextraction is routinely used. For derivatization, silylation and methoxylation are used for alcohol and aldehyde, respectively.

#### 1.4.3. Quadrupole Time-of-Flight (Q-TOF) Mass Spectrometry

To determine the chemical structure of unknown PFAS species, an untargeted quantification using a Q-TOF mass spectrometer was used (Table 1). Q-TOF allows the identification of unique compounds in a variety of samples, such as soil, drinking water, food, and human plasma [15]. Q-TOF is mainly used for the identification of unknown emerging PFAS. For volatile PFAS assay, GC-Q-TOF is accepted in a recent study [19].

#### 1.4.4. Combustion Ion Chromatography

By increasing the number of emerging PFAS, there is an increasing demand to estimate the total concentration of PFAS. Current analytical methods employ LC-MS/MS for quantification and LC-Q-TOF for non-targeted screening of PFAS compounds. For LC-MS/MS assay, the analyte needs to be specified before quantification by LC-MS/MS with an appropriate internal standard. Alternatively, a non-target assay allows for the identification of unknown PFAS. In this case, it is impossible to quantify them due to a lack of proper internal standards. Thus, to estimate the total PFAS concentration, such a specific assay is needed.

Combustion ion chromatography (CIC) measures fluoride in the sample (Table 1). Depending on the sample preparation, a variety of methods have been proposed (Table 3). The total fluorine (TF) method quantifies fluoride in the sample without any sample pretreatment. Thus, endogenous inorganic fluoride might be predominant in liquid samples such as water, wet soil, and human plasma. The absorbable organic fluorine (AOF) method measures activated carbon-absorbable fluoride by CIC. In this case, the analyte essentially contains organic compounds. The extractable organic fluorine (EOF) method is similar to AOF, but EOF uses a variety of extraction procedures such as weak anion exchange. In EOF, the nature of target compounds would become more selective compared to AOF.

To determine what percentage of total fluoride is attributable to PFAS remains an open question. To achieve this, both EOF-derived total fluoride concentration and LC-MS/MS-derived specific PFAS concentration were compared [48]. As a result, PFAS accounted for only 1/5–1/8 in the EOF. A subsequent study revealed that most EOF was found to be from pharmaceuticals and pesticides [48]. In fact, a similar calculation has been made in separate studies [51,52]. Given that emerging PFAS usually have lower concentrations than legacy PFAS, such as PFOS, the usefulness of the EOF assay is expected in the future, particularly after legacy PFAS mitigation proceeds.

#### 1.4.5. Comparison of Methodology

Currently available methodology for PFAS measurement is summarized (Table 4). Because PFAS are found in many matrices such as water, soil, food, human plasma, and industrial products, this author chose serum as a matrix for subsequent discussion. LC-MS generally provides a lower limit of detection or quantitation. It is a robust assay when matrix effects, such as ion suppression, are properly considered. GC-MS appears to be less sensitive compared to LC-MS, possibly because of the volatility and stability of target compounds. Apparently, these low limits of detection can be achieved when a proper internal standard is available. HRMS is used for non-target analysis with a reasonably low limit of detection, possibly due to an elimination of contaminated interference compounds. CIC has the advantage of total fluorine quantification but is less sensitive. This is because all mass spectrometric quantification is performed under vacuum, while the detection of CIC uses an electrical conductivity detector.

## 2. Distribution of PFAS

**Figure 5:**
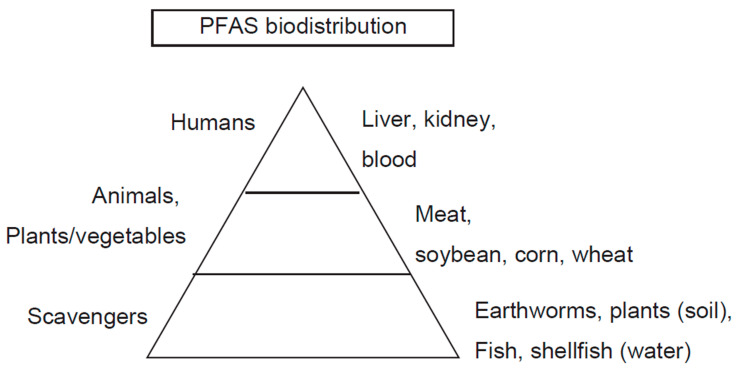
PFAS biodistribution.

PFAS have been synthesized in almost all industrialized countries, such as the U.S., EU, Canada, Japan, Australia, China, and India. As a result, legacy PFAS have accumulated almost everywhere. Historically, PFAS had been found in soil, especially that which is close to chemical industries, as well as the places that have been previously used for airports, firefighting areas, and military areas [33] (Table 5). Water, such as river water, groundwater, and seawater, is another source of PFAS. Once PFAS accumulate in these waters, it is highly anticipated that fish and shellfish will be highly contaminated [57]. In humans, the intake of water and food is a major source of PFAS accumulation in the body (Figure 5). This leads to the regulation of PFAS concentrations in drinking water in some industrialized countries.

### 2.1. Soil/Environmental Water

Since PFAS were first synthesized more than eight decades ago, PFAS are normally found everywhere at various levels [1] (Table 5). Perhaps, based on existing data, the U.S. is the country that has most extensively measured PFAS levels. In addition to previously described areas, rivers might show high PFAS concentrations when such places are located upstream. From the viewpoint of the conservation of land and water, a wastewater treatment plant (WWTP) is another source of PFAS. Another area where PFAS are highly detectable involves landfills; in these cases, whether such a landfill might contain high PFAS or not is apparently clear.

### 2.2. Drinking Water

The levels of contaminants, such as chloride, pesticides, metals, and other chemicals in drinking water, have been highly regulated by every country’s authority. PFAS’ concentration in drinking water is consistently regulated in some countries [42,43,79]. In some studies, 45% of tap water in the U.S. contains at least one type of PFAS [80]. The regulatory threshold of PFAS in drinking water depends on each country, with its own criteria [42,43]. PFAS levels in drinking water might be affected by upstream water sources. Furthermore, PFAS levels in groundwater could also affect those in drinking water. After the effort of PFAS mitigation in water sources, such as soil, rivers, and landfills, PFAS levels, particularly PFOS, gradually decreased in the U.S. and Japan [2,81].

### 2.3. Food

Food is another source that potentially elevates PFAS in humans through the food chain. Like soil and drinking water, PFAS accumulation is also consistently found in many places. Lifetime-exposed dairy cattle contain high PFAS levels [82]. In aquatic living things, high PFAS levels in clams have been reported [67,68]. Consequently, potential PFAS contamination might reach freshwater fish [69]. In the EU, a study reported high PFAS accumulation in eggs [71]. In Japan, PFAS accumulation in a Pacific cod has been reported [73]. In this particular case, these fish were caught at sea, far from mainland Japan; thus, severe PFAS contamination of this marine area was a concern.

### 2.4. Biodistribution in Humans

#### 2.4.1. Blood

Biomonitoring of PFAS in humans has been performed in many countries [2,33,83,84,85] (Table 6). Numerous studies have consistently demonstrated an increase in legacy PFAS concentrations before their phase-out. The half-life of PFAS ranges from one year to a decade in the order of PFHxS > PFOS > PFOA [86]. Importantly, a U.S. study demonstrated a decline in plasma PFAS, particularly PFOS, clearly demonstrating that an accumulated PFAS could be excreted in humans regardless of either endogenous or exogenous metabolic systems [2,81]. Food and water are two major routes of PFAS intake in humans. Fish and seafood are considered major sources of PFAS in the Japanese population.

#### 2.4.2. Liver

The liver is a major organ that metabolizes exogenous chemicals through a variety of reactions, such as oxidation, reduction, and conjugation. Consistently, a high concentration of PFOS was detected in the liver, followed by PFOA, PFNA, and PFHxS in humans [108]. Concerning the concentration of PFAS detected in the specimens, a comparison of this and a previous result obtained a decade ago, when PFAS production was not yet prohibited [99], revealed a marked reduction in newer specimens. Concerning PFAS species preference, this is also consistent with previous results in mice: it is known that long-chain PFCAs, such as PFOA (C8), PFNA (C9), PFDA (C10), and PFUnDA (C11), accumulate in the liver and plasma, while shorter-chained PFCAs, such as PFHpA (C7) and PFHxA (C6), are essentially excreted into the urine [81].

#### 2.4.3. Kidney

Similar to the liver, the kidney is another organ where PFAS compounds tend to accumulate at high concentrations [108,109]. Organic anion transporter 4 has been the best-examined transporter of PFAS in the kidneys. Physiologically, the kidney plays a key role in PFAS excretion [81]. Specifically, PFOS and PFNA showed the highest renal excretion, with decreasing excretion in the longer PFCA. Excretion of PFAS in humans is two orders of magnitude slower than that in rats, possibly because of the high binding of PFAS to renal protein.

## 3. Clinical Manifestations

Accumulating evidence demonstrates that PFAS, particularly longer-chained PFAS, tend to remain in humans in contrast to rodents [2,81]. This suggests that humans are likely to exhibit several manifestations (Table 6). Among them, dyslipidemia, attenuated immune response, and renal disorder are described below.

### 3.1. Dyslipidemia

**Figure 6:**
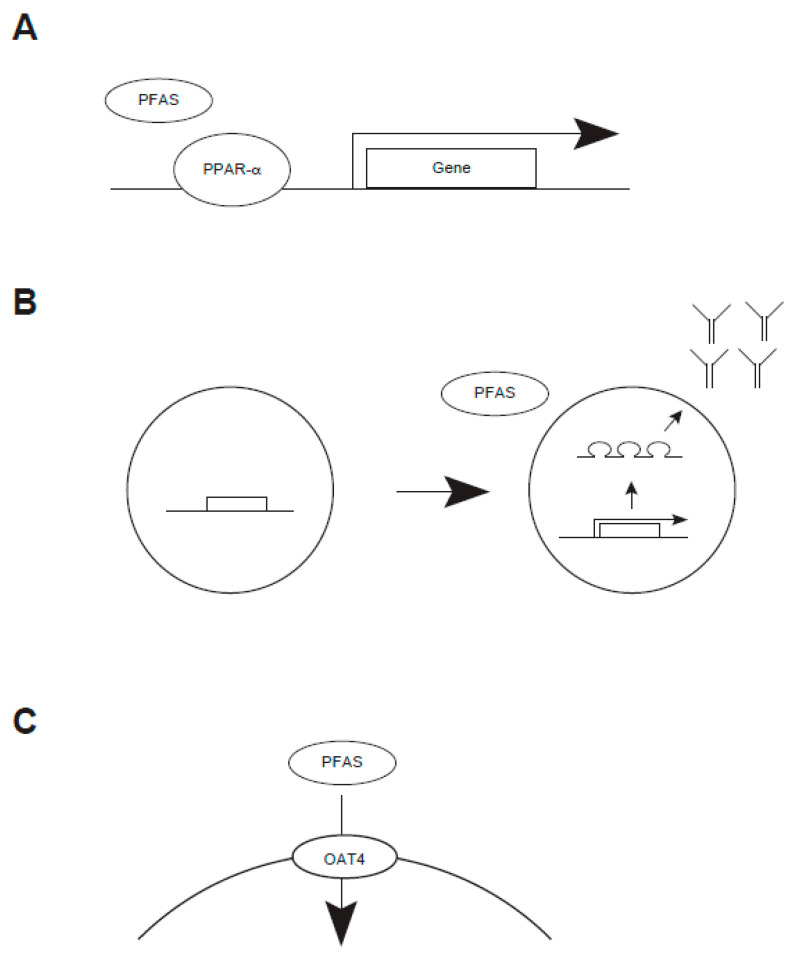
Biochemical action of PFAS (**A**–**C**).

An earlier study reported that total cholesterol and LDL cholesterol were inversely correlated with PFAS concentrations, such as PFOS [110,111,112]. Such a trend might be found regardless of age and sex, raising the possibility that PFAS would modulate cholesterol accumulation in these populations in the same manner. On a molecular basis, several studies have suggested that PPAR-α may be involved in this bioprocess [81,113] (Figure 6). While an increase in cholesterol due to PFAS has been suggested in humans, a recent review concluded that PFAS accumulation appeared not to be linked to atherosclerosis [112]. While PPAR receptors accommodate a relatively wide variety of substrates, short-chained PFAS might not activate this receptor as effectively as long-chain ones do.

### 3.2. Attenuated Immune Response

An epidemiological study suggested that a high-PFAS exposure group seemed to be highly susceptible to COVID-19 [42,43]. Emerging evidence suggests a potential link between this and impaired antibody production [114] (Figure 6). Earlier studies reported the mortality of COVID-19-related events [94,97]. Subsequent studies elucidated that the level of antibodies against COVID-19-derived viral antigens was decreased in PFAS-exposed individuals [99,101]. Research on children with allergies found an unexpected result: those exposed to PFAS compounds demonstrated reduced sensitivity to food allergen reactions compared to unexposed children [115]. The role of PFAS on immune response might be modulated, at least in part, by NF-κB, PPAR, calcium signaling, and cytokine-mediated signaling; thus, future study will be required [116]. Whether the immunological outcome could be modulated by the chain length of PFAS remains uncertain.

### 3.3. Renal Disorder

Studies have shown that high plasma PFAS concentrations, such as PFOS, are correlated with renal disorder [7,117] (Table 6). A possible mechanism raises a potential link between PFAS and renal transportation through human organic acid transporter 4 [113] (Figure 6). One study reported that exposure to PFAS increased the severity of chronic kidney disease, while their mortality remained unchanged [118]. Increasing PFAS chain length has been shown to increase its OAT4-binding activity in legacy PFAS (PFCA and PFSA) and an emerging PFAS (FTSA) in vitro and in silico [119].

## 4. PFAS Mitigation

### 4.1. Removal Technology

**Figure 7:**
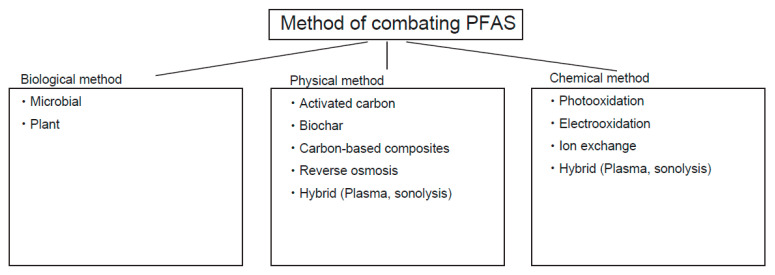
Methods of combating PFAS.

As mentioned, PFAS are less susceptible to chemical degradation. The bond dissociation energy between a C-F bond is approximately 130 kcal/mol, higher than that of a C-H bond (110 kcal/mol) [120]. As mentioned, this chemical property is suitable for numerous applications of PFAS, such as AFFF, nonsticking chemicals, and other applications. However, this chemical property is problematic when its degradation needs to be discussed [1] (Figure 7).

#### Physical Removal of PFAS

Physical adsorption processes, including activated carbons, biochar, carbon nanotubes, and carbon-based composites, have been evaluated for the removal of PFAS from various complex water matrices [60] (Table 7). All of these methods are essentially linked to carbon with different properties. Activated carbons are made from coal or oil-derived materials that have a high surface area for higher absorption of small molecules, such as PFAS. Biochar is also made of carbon from wood-derived charcoal with a high surface area and different chemical properties. Activated carbons are made at higher temperatures (~900 °C) compared to biochars (~300 °C). Activated carbons can be functionalized with additional chemical groups to enhance their properties, whereas biochars generally cannot undergo such modifications. Activated carbons and biochar are significantly more affordable than carbon nanotubes.

Carbon-based composites are crude materials made with biochars by fermentation with multiple bacteria before use. For binding these carbon materials to PFAS, the most important interaction involves the electrostatic interaction between carbons through π–π interaction. Ionic interactions could modulate PFAS binding with carbons. Similarly, hydrophilic interactions, such as hydrogen bonding, play a role in binding. Among these techniques, activated carbon and biochars have been shown to be effective for PFAS mitigation in soil. Also, carbon-based composites have been increasingly attracting attention, and as a result, some of them have been commercialized [123,124,125]. Ion exchange and reverse osmosis are preferably used for PFAS mitigation in water [115,121]. Phytoremediation is a new technique that uses plant-associated microbes in roots for PFAS absorbance [121].

### 4.2. Destruction Technology

#### 4.2.1. Biodegradation

Based on previous studies, it was initially found that PFOA was more susceptible to biodegradation [126,127] (Table 8). The mechanism of biodegradation is usually studied by its decomposition products. In PFOA, shorter-chained PFCA species were detectable. This raises the possibility of the elimination of CO_2_, followed by the formation of carboxylic acid (reviewed in [128]). Aerobic bacteria, such as *Pseudomonas*, *Delftia acidovorans*, and *Acidimicrobium* sp. strain A6, were well-studied cases for PFAS biodegradation. More recently, PFOS-degrading enzymes in soil were subsequently identified in multiple cases, suggesting a consistent outcome that PFOS-degrading products were also found in a non-target study.

#### 4.2.2. Chemical Degradation

##### Photocatalysis

In contrast, PFOS degradation has been studied based on chemical degradation, including photo-oxidation and electro-oxidation, as the major chemical reactions [136,137] (Table 8). These reactions convert CF_2_-SO_3_^−^ to a C-centered radical by eliminating SO_3_^−^ by reduction, followed by the formation of a short-chained C-centered radical under anaerobic oxidation [138,139]. The Fenton reaction and ammonium persulfate-mediated oxidation were well studied for PFOS degradation. Under aerobic conditions, the termination of peroxyl radicals results in the generation of alkoxyl radicals and molecular oxygen, followed by the formation of alcohol. In fact, this reaction might apply to PFOA; in this case, the elimination of carbon dioxide occurs as a product of the initial reaction.

FTCA, fluorotelomer carboxylic acid; FTS, fluorotelomer sulfonates; PFOA, perfluorooctanoic acid; PFOS, perfluorooctanesulfonic acid; UV, ultraviolet.

##### Plasma-Mediated Degradation

Generation of plasma leads to PFAS degradation in aqueous solution [140]. Activated electrons and free radicals react with PFAS, leading to the decomposition of C-F bonding. This technique is best applicable to water and equivalent clean samples.

##### Sonolysis

Sonolysis irradiates high-frequency sonic waves into a PFAS-containing solution, generating the formation and decay of fine air bubbles [141]. Under this condition, the PFAS, a surfactant, accumulates at the interface between air and water. In these conditions, C-F bonding of PFAS might be dissociated upon high temperature and free radicals when air bubbles decay. The associated concerns of this method depend on frequency, power, and temperature. Optimization of these parameters is better carried out by AI today.

#### 4.2.3. Emerging Methods: Hybrid Treatments

Hybrid treatment of PFAS degradation usually performs PFAS’ physico-chemical separation/concentration followed by degradation [142]. Physical separation includes membranous separation, absorption, and foam fractionation. Generally, concentration of samples prior to PFAS degradation increases its efficiency, leading to cost reduction. Membranous separation yields a clear solution that can be easily concentrated, leading to a cost-effective destruction process. The adsorption process can be easily combined with the current water purification process in a water purification facility. The foam-mediated phase separation technique applies wastewater, which accumulates highly concentrated foams. Such concentrated foam, then, is going to be degraded by sonolysis as described above.

### 4.3. Summary of the Latest Policy Frameworks (EU, US EPA) with Implications for Monitoring

#### 4.3.1. EU

The EU aims for a stepwise elimination of PFAS. The European Chemicals Agency is now proposing restricted use of PFAS for industrial application. For drinking water, the upper limit of total PFAS and of 20 PFAS compounds is 500 ng/L and 100 ng/L, respectively [143]. Development of PFAS alternatives and their degradation techniques is currently prioritized.

#### 4.3.2. US EPA

The US EPA regulates PFOA and PFOS concentrations at very low levels (4 ng/L). Similar to the EU, the US EPA also encourages the development of PFAS mitigation procedures. Importantly, the government expresses an intention to ask PFAS-manufacturing companies to pay the cost to obtain PFAS-free water for American citizens.

## 5. Perspectives

The concentration of legacy PFAS has been regulated in the U.S. and the EU [79]. Tokranov A.K. et al. measured legacy PFAS concentrations in wells and mapped this distribution across U.S. aquifers [79]. These authors demonstrated that this study allowed for the prediction of PFAS levels in each area, providing the possibility of whether any additional remediation might be required. Washington, JW et al. studied chloroperfluoropolyether carboxylates, a series of emerging PFAS in New Jersey, using non-target mass spectrometry [144]. These authors tentatively identified ten compounds, of which nine congeners contained (CF_2_)_7_ or a longer chain. It was found that the shorter-chain PFAS were highly detectable in far more distant areas; thus, these authors concluded that shorter-chain PFAS were mainly delivered atmospherically.

Selective detection of PFAS has largely been achieved by conventional chromatography-coupled mass spectrometry. Recently, a variety of sophisticated detection methods have been reported. Ion mobility mass spectrometry allows for the acquisition of the collision cross-section, which depends on the tertiary structure of low-molecular-weight compounds. In other words, a globular compound can be identified separately from a flatter compound by a difference in drift time. Kirkwood-Donelson et al. used this technique on many uncharacterized PFAS-related compounds [145]. Photoionization mass spectrometry, in theory, ionizes an analyte compound to give rise to fluorocarbon radicals. Xu M-G et al. successfully applied this technique for fluorinated compounds to demonstrate the formation of fluorocarbon radicals at high temperatures (400–950 °C). This photoionization technique generates characteristic fragmented ions that will never appear in electrospray and atmospheric pressure ionization [146]. Cyclodextrin-mediated molecular recognition is often used for molecular recognition and is studied for the single-molecule detection of DNA and peptides. Wei X et al. used cyclodextrin-mediated host–guest chemistry for PFAS-profiling at low levels [120]. Apart from the mass spectrometric technique, this nanopore-based PFAS assay is affordable; thus, its expansion into routine PFAS monitoring is highly expected.

Thousands of PFAS, as well as their precursors/metabolites, are known to accumulate and are characterized by non-targeting mass spectrometry. For the rapid identification of uncharacterized compounds, Wang X et al. developed novel machine learning software, characterizing its unreported structure by mass spectral data [147]. These authors originally developed a PFAS_ID module that allows the extraction of highly fluorinated compounds from spectral data. To demonstrate the efficacy of this system, fluorinated compounds were screened in wastewater. As a result, substantial species of unreported PFAS were detected globally in both legacy and emerging PFAS.

## 6. Conclusions

A large amount of research evidence on both legacy and emerging PFAS has been available now. Individual research goals have been reasonably achieved by many researchers’ efforts. Below is a list of remaining factors to be considered in the future:

- Global method harmonization;
- Precursor-to-product transformation assessment;
- Development of fluorine-free alternatives.

Global method harmonization is always problematic in other research areas, including clinical, forensic, and food chemistry. This could originate from the target PFAS to be regulated. For example, the quantification of which emerging PFAS compounds need to be regulated still needs discussion. EOF provides a good measure for PFAS, while its sensitivity is normally low. Radical-mediated degradation of a PFAS compound leads to a smaller PFAS radical. In this reaction, a variety of small compounds such as PFBA and TFA will be produced. These are volatile compounds that will evaporate into the air. The balance between precursor and product is usually not consistent, because the volatile PFAS are quantified by GC-MS, while LC-MS is used for the measurement of legacy PFAS and their associated degrading compounds. Finally, chemical industries are studying fluorine-free alternatives [148]. The current major area of PFAS consumption includes semiconductors and batteries. Continuous efforts to search for such alternative chemicals are urgently required.

## Figures and Tables

**Table 2:** Reported emerging PFAS in the environment and humans.

Year	Compound	Sample	Country	Reference
2020	Gen-X, F-53B, FC-98, and short-chain PFAS	Soil	China	[4]
2022	7:3 FTCA	Wildlife birds and eggs	Svalbard/ Norway	[9]
2022	F-53B, 8:2 Cl-PFESA	Indoor dust	China	[11]
2022	Gen-X	Freshwater fish	Czech	[34]
2024	Gen-X	WWTP	U.S.	[35]
2024	Gen-X, 6:2 FTS	Smallmouth bass	U.S.	[36]
2024	Gen-X, F-53B, PFBA, and PFBS	Seawater	China	[8]
2025	Gen-X	Drinking water	Australia	[23]
2025	F-53B	River water	Korea	[37]
2025	Gen-X	Biosolid	Ireland	[38]
2025	Gen-X, PFBA, PFHxA	Serum	Canada	[39]

Cl-PFESA, chlorinated polyfluoroalkyl ether sulfonic acids; F-53B (6:2 Cl-PFAES), 2-(6-chloro-1,1,2,2,3,3,4,4,5,5,6,6-dodecafluorohexoxy)-1,1,2,2-tetrafluoroethanesulfonic acid (CAS-RN: 756426-58-1); FC-98, potassium perfluorobutanesulfonate (CAS-RN: 29420-49-3); Gen-X, azane;2,3,3,3-tetrafluoro-2-(1,1,2,2,3,3,3-heptafluoropropoxy)propanoic acid (CAS-RN: 2037-80-3); PFBA, perfluorobutane acid (CAS-RN: 375-22-4); PFBS, perfluorobutane-1-sulfonic acid (CAS-RN: 375-73-5); PFHxA, perfluorohexanoic acid (CAS-RN: 307-24-4); WWTP: wastewater treatment plant.

**Table 3:** Comparison of method and purpose among TF, AOF, EOF, and TOP assay.

Method	Purpose	Analyte	Sample Work-Up	Detection Method	Reference
TF	Total fluorine	Fluoride	None	CIC	[50]
AOF	Absorbable organic fluorine	Fluoride	Activated carbon	CIC	[50]
EOF	Extractable organic fluorine	Fluoride	SPE	CIC	[50]
TOP	Total oxidizable precursor	Oxidant-reactive PFAS precursors	SPE	LC-MS/MS	[50]

AOF, Absorbable organic fluorine; CIC, combustion ion chromatography; EOF, Extractable organic fluorine; SPE, solid phase extraction; TF, Total fluorine; TOP, Total oxidizable precursor.

**Table 4:** Quantification procedure for PFAS.

Method	Assay	LOD/LOQ (μg/L)	Matrix	Matrix Effect	Effect	Robustness	References
LC-MS	Target analysis, TOP assay	0.02–0.2	Serum	Yes	Ion suppression	Yes	[12,53]
GC-MS	Target analysis	0.05–1.80	Serum	Yes	Ion suppression	Yes	[12,54]
HRMS	Non-target analysis	0.1–1.0	Serum	Yes, but relatively small	Ion suppression	Yes	[12,55]
CIC	TF, AOF, EOF	6–9	Serum	Yes	Overlapping of chloride ion	Yes	[50,56]

HRMS, high resolution mass spectrometry.

**Table 5:** PFAS levels in various specimens.

Specimen	Country	Sample	References
Soil	U.S.	Landfill	[58]
	U.S.	Farm field	[59]
	U.S.	Soil	[60]
	Germany	River water	[61]
	Australia	Soil	[62]
	Japan	Soil	[63]
	India	Sediment	[42]
	China	River water	[61]
Drinking water	U.S.	Surface water + groundwater	[64]
	EU	Surface water + groundwater	[65]
	Australia	Tap water + bottled water	[43]
	Japan	Surface water + groundwater	[66]
	India	Groundwater	[42]
Food	U.S.	Total diet	[14]
	U.S.	Seafood	[67]
	U.S.	Food	[68]
	U.S.	Food	[15]
	U.S.	Freshwater fish	[69]
	U.S.	Fish	[70]
	EU	Egg	[71]
	Australia	Livestock	[72]
	Japan	Pacific cod	[73]
	India	Fish	[42]
Human plasma/serum	U.S.	Plasma	[69,74]
	U.S.	Blood	[2]
	Germany	Blood	[75]
	Australia	Human serum	[76]
	Japan	Maternal serum	[77]
	India	Water, human serum	[42]
Specimen from an uncivilized area	Svalbard/Norway	Meltwater	[78]
	Germany	Wildlife	[10]

EU, European Union; U.S., United States.

**Table 6:** Major manifestations of PFAS-exposed humans.

Manifestation	Year	Country	Population	Reference
Dyslipidemia	2014	Norway	Pregnant women (n = 891)	[87]
	2022	U.S.	Age range 6–86 years old (n = 326)	[88]
	2023	U.S.	Women aged 45–56 years old (n = 1130)	[89]
	2024	China	Non-fasted individuals (n = 575)	[90]
	2025	Korea	Adolescents aged 12–17 years old (n = 824)	[91]
	2025	U.K.	A healthy unselected population of twins (n = 2069)	[92]
	2025	Canada	Pregnant women (n = 282)	[5]
	2025	Norway	Non-diabetic participants (n = 145)	[93]
	2025	Canada	Adult males with elevated cholesterol (n = 72)	[39]
Impaired immune response	2020	Denmark	SARS-CoV-2-infected subjects aged 30–70 years old (n = 323)	[94]
	2021	Sweden	Age- and sex-standardized adult population (control district n = 898; contaminated district n = 239)	[95]
	2021	Italy	PFAS-positive area (n = 187,375); control area (n = 4,750,548)	[96]
	2022	U.S.	Age-matched comparison	[97]
	2022	U.S.	Aged ≥ 20 years old (n = 415)	[98]
	2023	U.S.	Unvaccinated (n = 153); vaccinated (n = 860)	[99]
	2023	U.S.	Pregnant women (n = 72)	[100]
	2024	Denmark	Aged 50–69 years old (n = 477)	[101]
	2025	U.S.	Pregnant women (n = 59)	[102]
Exacerbated CKD	2019	U.S.	Adults ≥ 20 years old (n = 8220)	[103]
	2024	U.S.	Young adults (n = 78)	[104]
	2024	U.S.	Adults with CKD (n = 3239); no CKD (n = 10,648)	[105]
	2025	Sweden	All aged 70 years old at baseline, 50% females (n = 997)	[106]
	2025	China	Aged ≥ 20 years old (n = 1503)	[20]
	2025	China	Adults (n = 2801)	[107]

CKD: chronic kidney disease; U.K., United Kingdom.

**Table 7:** Engineering techniques for PFAS mitigation.

Method	Absorption	Cost	Decomposition	Reference
Activated carbons	++	++	No	[60]
Biochar	+++	+	No	[60]
Carbon nanotubes	+	+++	No	[60]
Carbon-based composites	+++	+	Yes	[60]
Ion exchange	+	+++	No	[115]
Reverse osmosis	+	+++	No	[121]
Phytoremediation	+	++/+++	Yes	[122]

+++ very strong, ++ strong, + modest.

**Table 8:** Degradation of PFAS by biological and chemical procedures.

Method	Substrate	Catalyst/Materials	References
Microbial	PFOA	*Pseudomonas*	[129]
	PFOA, PFOS	*Delftia acidovorans*	[130]
	PFOA, PFOS	*Acidimicrobium* sp. strain A6	[131]
	PFOS	*Pseudomonas aeruginosa*	[132]
	PFOS	*Pseudomonas plecoglossicida* 2.4-D	[131]
	FTCA	Hyphomicrobium, Methylorubrum, and Achromobacter	[133]
	FTS	*Dietzia aurantiaca*	[134,135]
Photo-oxidation	PFOS	UV	[136]
Electro-oxidation	PFOS	Boron-doped diamond and mixed metal oxide	[137]

## Data Availability

Data sharing is not applicable.

## Abbreviations

AFFF	aqueous film-forming foam
AOF	absorbable organic fluorine
CIC	combustion ion chromatography
EOF	extractable organic fluoride
F-53B	(2-((6-chloro-1,1,2,2,3,3,4,4,5,5,6,6-dodecafluorohexyl)oxy)-1,1,2,2-tetrafluoroethanesulfonic acid)
GC-MS	gas chromatography–mass spectrometry
Gen-X	hexafluoropropylene oxide-dimer acid
HFPO-DA	hexafluoropropylene oxide-dimer acid
LC-MS/MS	liquid chromatography–tandem mass spectrometry
PFAS	Per- and polyfluoroalkyl substances
PFCA	perfluorocarboxylic acid
PFHxS	perfluorohexane sulfonate
PFOS	perfluorooctane sulfonate
PFOA	perfluorooctanic acid
PFSA	perfluorosulfuric acid
Q-TOF	quadrupole time-of-flight
QuEChERS	Quick, Easy, Cheap, Effective, Rugged, Safe
TF	total fluorine
TOP	total oxidizable precursor
WWTP	wastewater treatment plant
